# Physical and neural entrainment to rhythm: human sensorimotor coordination across tasks and effector systems

**DOI:** 10.3389/fnhum.2014.00576

**Published:** 2014-08-01

**Authors:** Jessica Marie Ross, Ramesh Balasubramaniam

**Affiliations:** Sensorimotor Neuroscience Laboratory, Cognitive and Information Sciences, University of CaliforniaMerced, CA, USA

**Keywords:** neuroentrainment, sensorimotor coordination, timekeeping, force control, state estimation, rhythm

## Abstract

The human sensorimotor system can be readily entrained to environmental rhythms, through multiple sensory modalities. In this review, we provide an overview of theories of timekeeping that make this neuroentrainment possible. First, we present recent evidence that contests the assumptions made in classic timekeeper models. The role of state estimation, sensory feedback and movement parameters on the organization of sensorimotor timing are discussed in the context of recent experiments that examined simultaneous timing and force control. This discussion is extended to the study of coordinated multi-effector movements and how they may be entrained.

## Introduction

Entrainment is a broadly used term that refers to the process of alignment between systems. In the context of human behavior and physiology, entrainment refers to the adaptive function by which we voluntarily or involuntarily synchronize our brains and bodies to the environment. As this encompasses the coupling of brain and behavior, this phenomenon is often referred to as neuroentrainment. A commonly used paradigm in neuroentrainment research is to study the temporal relationship between the body and the rhythmic stimulation in the environment (Balasubramaniam, 2006; Keller and Repp, 2008). This temporal coupling between the body and rhythmic stimulation has been used to study: (1) the variability, stability and adaptability of the entrainment; (2) the coordination between multiple effectors and the environment; and (3) the neural basis of rhythm and rhythmic timekeeping. While the former two have benefited greatly from development in dynamical systems models, the latter has been very successful in recent years due to advances in brain imaging.

Entrainment to the environment is possible with any sensory modality: auditory, visual, tactile or vestibular. However, synchronization to sound appears to be temporally more precise and accurate, especially compared to visual entrainment (Hove et al., 2013a). This is not to say that visual entrainment is in any way less important. The time scales of its operation and the feedback loops that work in error detection and correction end up being different from those seen in entrainment to auditory stimuli. Visuo-motor synchronization can improve when the stimuli are moving targets, but there still is an advantage for auditory-motor synchronization (Hove et al., 2010, 2013b). In addition, the neural structures that subserve synchronization to visual and auditory stimuli differ, with auditory-motor synchronization activating more structures that are associated with internal rhythmic movement control (Jäncke et al., 2000). Taken together, all this evidence supports the idea that the coupling between the auditory and motor system is privileged, especially in the context of rhythm.

The sounds we hear influence our motor behavior. Normal healthy adults have low level and automatic responses to sounds (Rossignol and Melvill, 1976; de Manzano et al., 2010; Stupacher et al., 2013). This relationship between sounds and movement is prevalent throughout the lifespan. Infant vocalizations vary depending on the infants’ linguistic environments (de Boysson Bardies and Vihman, 1991), and attachment and aspects of cognition can be predicted by the degree of timing coordination between infants and their caregivers (Jaffe et al., 2001). People with Parkinson’s disease can use rhythmic sounds to improve their walking movements (Thaut and Abiru, 2010). Even passive listening to rhythmic stimuli activates motor regions of the brain (Grahn and Brett, 2007; Chen et al., 2008). Musicians in particular learn to harness this ability to synchronize with fellow musicians in an ensemble. Although sensory and motor timing mechanisms are often thought of as separate yet similar, there is evidence that they are might share a common neural mechanism (Meegan et al., 2000). The relationship between our auditory and motor systems can be capitalized on to influence motor behavior in positive ways, including to aid in motor development and rehabilitation (Hove et al., 2012).

In this review, we focus on sensorimotor coordination and neuroentrainment using two major theoretical approaches. In one approach, sensorimotor timing and entrainment are viewed as the product of a centrally controlled timekeeping mechanism. In another approach, entrainment is considered to be an emergent property that arises from the interaction between the body mechanics, distributed brain networks and environment (for review see Torre and Balasubramaniam, 2011). Here we review research from our own laboratory and others, dealing with sensorimotor coordination specific to rhythmic entrainment. This includes the influence of entrainment on individual effectors (such as the finger, arm, eye) or coupled effectors, (homologous, such as two fingers, and non-homologous neuromuscular structures, such as finger and eye) and the coordination of full body motion, such as the control of posture and balance.

Different types of motor behavior will also be reviewed, such as finger tapping, force production, and saccadic eye movements when entrained to an external metronome. The general consensus from our article is that the abovementioned approaches are not mutually exclusive and that both are equally relevant for the study of entrainment.

## Theories and models of voluntary timing

The body is a complex system that takes in environmental information, processes this information, and creates motor output. This motor output is complex because it involves numerous muscles and joints and all the configurations they can achieve. In other words, many dimensions must be taken into account to produce even a very simple motor output. In order to entrain to a stimulus train, this complexity needs to converge on the necessary dimensions in order to produce synchronized and controlled movement, taking into account motor delay and variability. The Wing-Kristofferson model tackles this problem as a process that involves a central timekeeper, or clock, that controls the timing intervals and the peripheral motor system that implements the signals from the timekeeper (Wing and Kristofferson, 1973). In this approach, time is represented centrally, independent of the motor system. According to this model and its underlying assumptions, timekeeping does not rely on feedback from the effectors and is relatively independent of the movements themselves.

**Figure 1:**
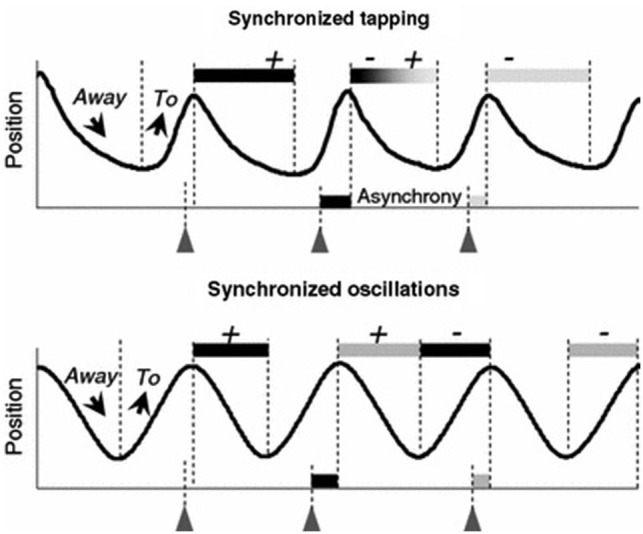
**Correlation patterns between the asynchronies and the two preceding and two following phases *To* and *Away* observed in synchronized tapping and oscillation tasks**. The *+* and *− signs* represent the positive or negative correlations between the phases and the related asynchrony. During discrete tapping the duration of a given *Away* phase is related to both the preceding and the succeeding asynchronies. During smooth finger oscillations, two consecutive asynchronies do not happen to be correlated with the same semi-cycle (reproduced with permission from Torre and Balasubramaniam, 2009).

However, recent evidence indicates that movement trajectories contribute to movement timing. Timed repetitive finger tapping has become an established method for studying motor entrainment with limited degrees of freedom. This task requires timing accuracy and period stability, often to a metronome. Finger movement trajectories in this paradigm demonstrate asymmetry, and this asymmetry is negatively correlated with timing accuracy, and decreases at higher tapping frequencies (Balasubramaniam et al., 2004). Specifically, higher velocity movements occur in the flexion cycle before each tap (to aid in synchronization with the beat) and lower velocity movements occur in the extension cycle after each tap (as a correction to maintain period accuracy). Further analysis has revealed that the movement trajectories contribute to the achievement of synchronized movement timing in two different ways. A description of this phenomenon is shown in Figure 1.

Discrete movements like tapping and continuous movements like circle drawing might not share the same timekeeping mechanism (Spencer et al., 2003). Neuropsychological evidence indicates that individuals with cerebellar damage, who show compromised timing skills in finger tapping tasks, do not exhibit similar error patterns in circle drawing. It has been suggested that continuous movements such as circle drawing have different properties than discrete movements. Timing in such movements has been hypothesized to be an emergent property of the interaction of the neuromuscular system with the environment.

Long-range correlations can be used to understand how points in a time series are related to the mean of that series. If two time series have similar long-range correlation patterns, this can support that similar mechanisms are at play. While individuals show consistent signatures of long-range correlational patterns in tapping and circle drawing, these patterns are not correlated across tasks (Torre et al., 2011). However, continuous and discrete movements have similar long-range correlations during synchronization to a metronome (Torre et al., 2010). Similarly, if a perceptual event is added to a continuous task (such as a tactile difference at one point in a circle drawing task), these continuous movements become more event-like in their nature (Studenka et al., 2012).

Collectively these results suggest that timekeeping cannot be described only as a centrally controlled mechanism or as an emergent property of the body mechanics and environment. The most realistic model for human timing should take both views into account. It is likely that timekeeping functions rely on a more distributed neural network whose dynamics might be observed in the activation of areas including the sensorimotor cortices, supplementary motor area (SMA), basal ganglia and the cerebellum (Molinari et al., 2003).

## Timing, force and state estimation

In tasks like playing the piano we control the force of the keystroke in addition to timing of the motor response itself (Goebl and Palmer, 2008). It has been argued that force and timing might be independent of each other and controlled by neurophysiologically distinct mechanisms (Keele et al., 1987; Pope et al., 2005).

State estimation is the process of determining a close approximation of the state of a system. In terms of human movement, this state might be the position of an effector, the movement of that effector through space, or the force being applied to something by that effector. This type of state estimation uses knowledge of the motor command and the predicted sensory consequences of an action. Accurate motor and sensory state estimation is necessary for appropriate motor control, and can be undermined by inaccurate information about the motor command or consequences of the action. In general, forces that we apply to ourselves seem weaker than external forces because of self-cancellation, a function of having an efference copy (Shergill et al., 2003).

While a good amount of previous research has focused on timekeeping processes, less is understood about whether movement parameters such as force show sequential dependencies when entrained. Therrien and Balasubramaniam (2010) examined the effect of timing constraints on repetitive unimanual force production sequences. In this experiment, either visual feedback of force produced or the auditory metronome was extinguished after a period of entrainment. In the continuation trials, a negative lag-1 autocorrelation in the inter-response intervals (IRIs) was observed as is commonly seen in motor timing tasks. However, removal of visual feedback after entrainment resulted in a systematic increase in mean force output through the course of the trial, resulting in positive lag-1 autocorrelation values. First, these results suggest a relative independence in the control of force and timing. Second, these findings suggest that accurate state estimation in this task requires visual feedback, in the absence of which the force errors increase with time. Forces produced at the fingertip being perceived as weaker also leads to a systematic, compensatory over-production of the magnitudes required.

**Figure 2:**
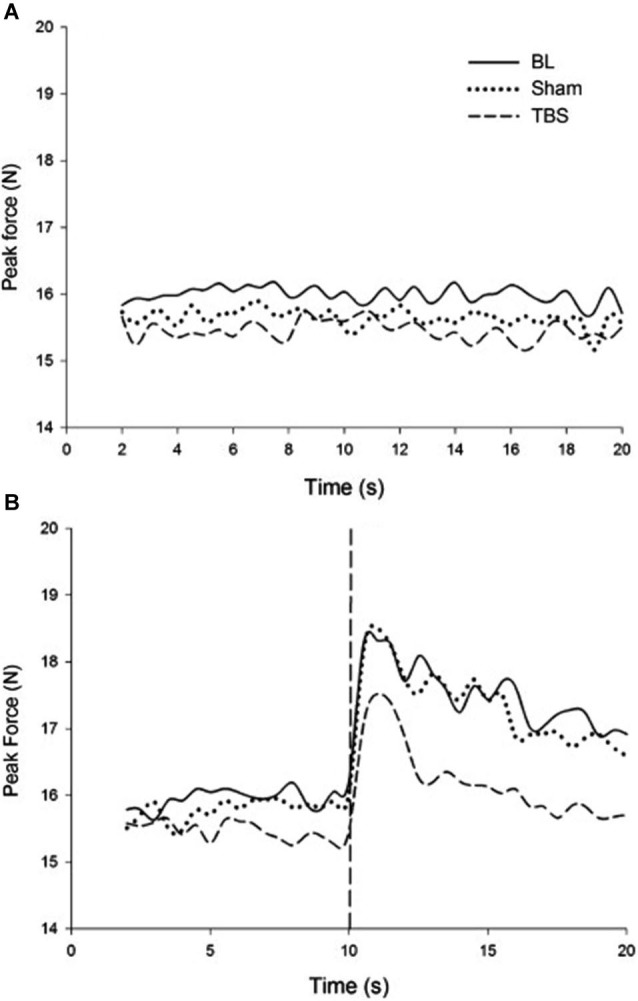
**(A)** Force produced during rhythmic entrainment. **(B)** Force produced during rhythmic entrainment when the entraining stimulus is extinguished at 10 s. It is readily noticed that the force escalation effect that is pronounced in the baseline and sham condition is reduced due to cTBS (reproduced with permission from Therrien et al., 2011).

In order to test the neurophysiological mechanism underlying this process, in a follow-up experiment, continuous theta-burst stimulation (cTBS) was applied to the motor cortex. cTBS is a relatively new technique that has been shown to reliably depress cortical excitability following stimulation. Applying cTBS to M1 has been shown to interrupt typical force attenuation (Voss et al., 2007). An explanation for this is that reducing cortical excitability of M1 through application of cTBS induces a discrepancy between the efference copy generated and motor output produced. In this experiment, the overproduction of forces following visual feedback removal was reduced after receiving cTBS. It was observed that mean peak force and its error were greater and more positive in the absence of visual feedback regardless of stimulation condition; however, the magnitude of increase was significantly reduced following cTBS compared with baseline and sham conditions. Thus, disrupting the state estimation process created a reduction in the force escalation effect reported in the earlier study (Therrien et al., 2011). Figure 2 describes these results in detail. cTBS over M1 during a bimanual force production task showed asymmetry between the rate of force increase in the two hands, suggesting that force control is not as symmetric in bimanual tasks as other types of motor control (Therrien et al., 2013).

This escalation effect is not unique to finger force production alone. In the absence of auditory feedback such an escalation effect is seen in the control of speech volume also, as can be readily demonstrated by individuals talking with headphones on. The automatic and involuntary increase in vocal intensity that speakers exhibit in a noisy environment is called the Lombard effect. Therrien et al. (2012) ran an experiment to study if the same mechanism of state estimation and sensory attenuation might underlie the expression of the Lombard effect. Participants vocalized phonemes entrained in time to a metronome, with visual feedback about the vocal volume levels. Auditory and visual feedback of their performance were manipulated or removed during the course of the trial after an entrainment phase. As predicted, the authors discovered an increase in voice volume in the absence of the visual feedback. Thus, providing a visual reference to calibrate somatosensory-based judgments of vocal intensity resulted in reduced expression of the Lombard effect. These results suggest that sensory attenuation mechanisms as seen in fingertip force production also play a role in the control of speech volume.

The escalation effect supports that state estimation influences motor production. This contests the assumption of the classic timekeeper models that motor control is independent of the motor system, and supports that timekeeping is influenced by information from the effectors. It has been proposed that pitch control in singing uses a similar state estimation process (Pfordresher, 2011). It would be interesting to see if this effect is seen in the control of pitch and volume in singing in the absence of auditory and visual feedback. Ongoing and future work in our laboratory will be addressing this question directly.

## Entrainment and coordination across effector systems

Tapping with two hands is different in many ways compared to tapping with one. The finding that tapping with two fingers on opposite hands exhibits reduced timing variability, as compared with tapping with only one finger is often referred to as the bimanual advantage (Helmuth and Ivry, 1996). It has been suggested that bimanual advantage results from the addition of either sensory (i.e., enhanced feedback) or cognitive (i.e., multiple timekeeper) processes involved in timing.

Crossing the arms impairs perception of tactile stimuli and modulates cortical activation following tactile stimulation (Shore et al., 2002). A recent study investigated the effect of crossing the arms on the clearly identified bimanual advantage (Studenka et al., 2014). In this experiment, participants tapped unimanually or bimanually with their arms crossed or uncrossed on a tabletop or in the air. A significant bimanual advantage was observed for the uncrossed, but not the crossed posture in finger tapping. However, removing tactile feedback from taps eliminated the bimanual advantage for both postures. These results suggest that the integration of internal (i.e., effector-specific) and external (i.e., environment-specific) information was impaired when crossing the arms. Furthermore, they suggest that successful multisensory integration is crucial to reducing timing variability during repetitive coordinated bimanual tasks. Other evidence that unambiguous sensory feedback at the effectors is important for sensorimotor integration and reduction of timing variability is provided by Keller et al. (2011). Thus, the bimanual advantage is not just a function of having more timekeepers at work.

Sensorimotor mechanisms underlying the control of eye movements are different, since they rely less on proprioception based state estimates. Saccadic eye movements are programmed to be very rapid and are considered to be relatively cost-free movements. The question of whether eye movements can be entrained to rhythmic stimuli is one that has not been addressed in many studies of timing. In a recent study of oculomotor timing, three methods of entrainment were used: saccade, continuous pursuit and discontinuous pursuit (Richardson and Balasubramaniam, 2010). When the stimulus train was extinguished after entrainment, subjects made saccadic eye movements at the entrained movement frequencies between two static targets. The pursuit entrainment conditions resulted in added clock and motor variance to the saccade entrainment. Thus the specific pattern of entrainment can have lasting influences beyond the time period of the entrainment itself. Jantzen et al. (2005) observed a similar entrainment specific effect, where participants were asked to make finger movements after being entrained to a rhythmic stimulus train, either in synchronization mode (in phase) or syncopation mode (anti phase) with an auditory metronome. Although in the continuation phase these movements were essentially similar, brain activation showed persistent patterns that were specific to the manner of entrainment.

Rhythmic entrainment can also create spatial interference in other effectors. Drawing a line with one hand and a circle with another make the line appear more like a circle and the circle more like a line (Franz et al., 1991). This phenomenon is often referred to as a magnet effect and can be seen even in amputee individuals that have phantom limbs (Franz and Ramachandran, 1998). Such unintentional movement interference is often seen when two limbs perform spatially dissimilar tasks, such as simultaneously patting one’s head and rubbing one’s belly. Recent work from our laboratory demonstrated a novel spatial interference effect between eye and hand movements, which are controlled by distinct central neural networks and descending motor tracts (Richardson et al., 2013). In one experiment, subjects performed finger tapping to a pacing stimulus while simultaneously making repetitive horizontal saccadic eye movements. The finger trajectory showed a lateral shift to the right when making a rightward saccade and to the left when making a leftward saccade. Said differently, vertical finger movements are unintentionally attracted in the direction of concurrently executed horizontal saccades when responses are planned or timed together. In a second experiment, participants performed finger tapping but were instructed to make reactive horizontal saccades following target jumps at unpredictable times. Here, the lateral shift that accompanied the saccades was weak and occurred only in the hand ipsilateral to the direction of the saccade. These results suggest that the recruitment of a common timekeeping mechanism can create spatial interference even in effectors that are innervated by distinctly different neurophysiological tracts.

## Conclusions and future directions

Collectively, this body of work shows that timekeeper theories of sensorimotor timing need to consider several important movement parameters related to effector position, sensory feedback and state estimation. A more complete theory of entrainment will be made possible by providing important linkages between the neural networks that make up “clock” like structures in the brain and the abovementioned movement parameters. While future research should also take on questions that deal with more complex problems on the side of entrainment parameters (such as metrical rhythm), equal attention should be paid to the coordination between various parts of the body that is achieved by entraining the human sensorimotor apparatus to these variables.

## Conflict of interest statement

The authors declare that the research was conducted in the absence of any commercial or financial relationships that could be construed as a potential conflict of interest.
